# Impact of sarcopenia in advanced and metastatic soft tissue sarcoma

**DOI:** 10.1007/s10147-021-01997-7

**Published:** 2021-07-27

**Authors:** Dennis Strassmann, Bennet Hensen, Viktor Grünwald, Katharina Stange, Hendrik Eggers, Florian Länger, Mohamed Omar, Patrick Zardo, Hans Christiansen, Christoph W. Reuter, Frank K. Wacker, Arnold Ganser, Philipp Ivanyi

**Affiliations:** 1 Klinik Für Hämatologie, Hämostaseologie, Onkologie Und Stammzelltransplantation, Medizinische Hochschule Hannover, OE 6860, Carl-Neuberg-Straße 1, 30625 Hannover, Germany; 2 Department of Radiology, Hannover Medical School, Hannover, Germany; 3 Clinic for Urology and Clinic for Medical Oncology, Interdisciplinary GU Oncology, University Hospital Essen, Essen, Germany; 4 Department of Radiotherapy, Hannover Medical School, Hannover, Germany; 5 Institute of Pathology, Hannover Medical School (MHH), Hannover, Germany; 6 Department of Orthopedics and Trauma, Hannover Medical School, Hannover, Germany; 7 Department of Cardiac, Thoracic, Transplantation and Vascular Surgery, Hannover Medical School, Hannover, Germany

**Keywords:** Sarcopenia, Soft tissue sarcoma, Smi, Skeletal muscle index, Survival, Body composition

## Abstract

**Introduction:**

Advanced or metastatic soft tissue sarcoma (a/mSTS) is associated with a dismal prognosis. Patient counseling on treatment aggressiveness is pivotal to avoid over- or undertreatment. Recently, evaluation of body composition markers like the skeletal muscle index (SMI) became focus of interest in a variety of cancers. This study focuses on the prognostic impact of SMI in a/mSTS, retrospectively.

**Methods:**

181 a/mSTS patients were identified, 89 were eligible due to prespecified criteria for SMI assessment. Baseline CT-Scans were analyzed using an institutional software solution. Sarcopenia defining cut-off values for the SMI were established by optimal fitting method. Primary end point was overall survival (OS) and secondary endpoints were progression free survival (PFS), disease control rate (DCR), overall response rate (ORR). Descriptive statistics as well as Kaplan Meier- and Cox regression analyses were administered.

**Results:**

28/89 a/mSTS patients showed sarcopenia. Sarcopenic patients were significantly older, generally tended to receive less multimodal therapies (62 vs. 57 years, *P* = 0.025; respectively median 2.5 vs. 4, *P* = 0.132) and showed a significantly lower median OS (4 months [95%CI 1.9–6.0] vs. 16 months [95%CI 8.8–23.2], Log-rank *P* = 0.002). Sarcopenia was identified as independent prognostic parameter of impaired OS (HR 2.40 [95%-CI 1.4–4.0], *P* < 0.001). Moreover, DCR of first palliative medical treatment was superior in non-sarcopenic patients (49.2% vs. 25%, *P* = 0.032).

**Conclusion:**

This study identifies sarcopenia as a prognostic parameter in a/mSTS. Further on, the data suggest that sarcopenia shows a trend of being associated with first line therapy response. SMI is a promising prognostic parameter, which needs further validation.

## Introduction

Despite improvements in surgical techniques, pathological understanding and medical treatment, patients with advanced or metastatic soft tissue sarcomas (a/mSTS) are mostly considered incurable [1]. Recent studies report a median overall survival (OS) of 10–18 months, and the 5-year survival rate for metastatic sarcoma is a mere 16% [2–4]. Also, despite several new tested drugs, as well as several discussed treatment strategies, during the past decade, no substantial improvement in outcome could be achieved for a/mSTS [5].

Guidelines generally advocate multimodal treatment at specialized centers with a multidisciplinary approach consisting of systemic medical treatment (CTx) and surgery (Sx) and/or radiotherapy (RTx) [6]. Hereby, nomograms are utilized in prediction of treatment outcome in localized disease or counseling towards adjuvant therapies [7, 8]. Also, clinical parameters like tumor localization or histopathological grade have been identified to significantly impact outcome and reflect critical parameters once counseling patients [9, 10]. In particular, once a patient enters the setting of a/mSTS disease stage, the prognosis becomes dismal and risk benefit evaluation of therapeutic aggressiveness is crucial to ensure optimal palliative benefit. However, hardly any valid tool is established for prognostication for therapy aggressiveness in a/mSTS patients.

Sarcopenia, which refers to the depletion of skeletal muscle has emerged as an independent predictor of outcome in a variety of different cancers [11–15]. Further on, it is easily accessible in routine CT-scans, wherein the skeletal muscle index (SMI) is measured for sarcopenia determination. Body composition parameters, like the SMI, can have an impact on risk assessment and clinical decision making in different cancer types. For instance, in advanced gastric cancer, sarcopenia was shown to be an independent prognostic factor for shorter OS and in colorectal cancer sarcopenia was also associated with reduced OS, as well as diminished progression free survival (PFS) [15, 16].

Therefore, body composition parameters are highly interesting to be evaluated in a/mSTS patients, who urgently need fast forward prognostication systems to avoid over- or undertreatment. To our knowledge sarcopenia has not been evaluated as prognostic marker for a/mSTS. Thus, the aim of this study was to analyze the impact of sarcopenia on outcome in a cohort of a/mSTS patients treated at a tertiary center, retrospectively.

## Materials and methods

### Patient data

181 patients over 18 years with a/mSTS treated at the Hannover Medical School between 12/1998 and 05/2016 were identified retrospectively. Patient data were extracted from the digital charts archive and the local clinical tumor registry. Data analysis was done in an anonymized manner in accordance with the declaration of Helsinki [17]. Patients with soft tissue sarcoma, who received palliative CTx with or without measurable disease were included. All soft tissue sarcoma histiotypes were permitted, except for Gastrointestinal Stroma Cell Tumors (GIST). Availability of an abdominopelvic CT scan of diagnostic quality within 14 days prior to start of CTx was required. CT scans were ineligible if readability was rendered impossible e.g. through metal artefacts or tumor invasion.

Clinical data, tumor- and treatment characteristics including age, sex, height, Eastern Cooperative Oncology Group performance status (ECOG), tumor grading, tumor size and localization, initial resection status and mode of metastatic spread as well as therapeutic lines and agents, additional surgeries and application of radiotherapy were obtained [18]. Mode of metastatic spread describes the occurrence of metastases in respect to initial diagnosis, synchronous spread was defined as diagnosis of metastatic disease within three months of initial diagnosis. Multimodal therapy was defined as at least one tumor related surgery (Sx) and/or tumor related radiotherapy (RTx) in addition to CTx. Predefined primary endpoint is overall survival (OS). Secondary endpoints are progression free survival (PFS), disease control rate (DCR) and overall response rate (ORR) of first palliative CTx, based on routine radiological judgment. Subgroup analysis of sarcopenic and non-sarcopenic patients were predefined.

### Assessment of sarcopenia

**Fig. 1:**
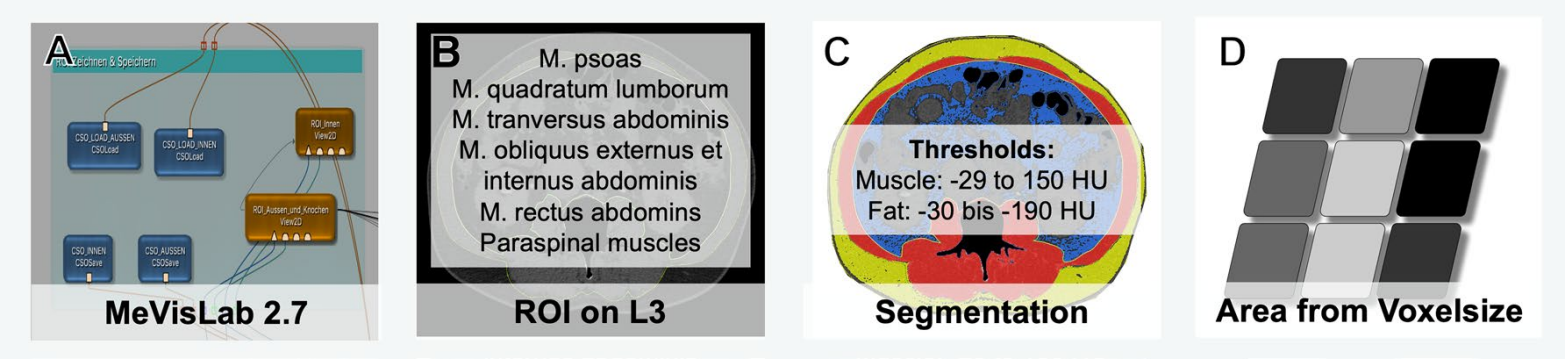
Workflow measurement setup of the body composition parameters analysis using MeVisLab 2.7

Sarcopenia was measured in pre-treatment CT scans using a specifically self-designed software-tool in MeVisLAB (MeVisLAB 2.7, Fraunhofer MEVIS, Bremen, Germany) for image evaluation by experienced radiology specialists (DS, BH). After importing axial DICOM images of the abdomen with a reconstruction interval of 5 mm and a standard soft reconstruction kernel into the MeVisLAB software, a cross sectional image on the level of mid-L3 vertebra showing both transverse processes was selected. The body compartments were segmented manually with a closed spline region of interest (ROI) in the selected slice. Within these areas, Hounsfield unit (HU) thresholds were defined for skeletal muscle with a range of −29–150 HU, according to consensus-based recommendations [19]. The derived area for muscle was calculated from the number of voxels and voxel size within the ROI (Fig. 1).

This area constituted the basis for calculating the skeletal muscle index (SMI), normalized by height, according to the formula:

$$SMI = \frac{{Area\;muscle\;L3\left( {cm^{2} } \right)}}{{height\left( {m^{2} } \right)}}$$

The gender specific cut-off values defining sarcopenia for the SMI were obtained by optimal fitting method from SMI values of the cohort [20]. This resulted in a sarcopenia defining cut-off for men of ≤ 44 and for women of ≤ 38.

### Statistical analysis

Data were analyzed with descriptive and inferential statistics. Categorical data were recorded as absolute frequencies, percentages and, if applicable, range. *P* values are based on different tests as appropriate. Shapiro–Wilk test was utilized to verify Gaussian distribution of the SMI. The cohort was stratified for sarcopenia, based on the appropriate SMI cut-off value, as mentioned above.

OS was defined as time from first palliative CTx until death or last visit. PFS was defined as time from first palliative CTx to progression or death by clinical or radiological judgement. Kaplan Meier analysis with log-ranks were administered for PFS and OS calculation. Univariate and multivariate Cox-proportional hazard regression analysis was employed to evaluate variables for their prognostic value. Variables with a *P* ≤ 0.2 in univariate analysis were subjected to a multivariate Cox-Regression analysis. Hazard ratios (HRs) and confidence intervals (CIs) were estimated. The alpha level for testing significance was set at *P* < 0.05. Statistical analysis was performed using SPSS v24 (IBM corp., Armonk, USA).

## Results

### Patient, tumor and treatment characteristics

89 (49%) of 181 a/mSTS patients were eligible for this retrospective analysis, according to predefined in- and exclusion criteria. The cohort consists of slightly more male than female patients (*n* = 49, 55.1%) with a median age of 60 (range (r), 20–79) years at diagnosis of a/mSTS. 70 (78.7%) patients showed a good performance status (ECOG < 1). At initial diagnosis 81 (91.0%) sarcomas were classified as deep according to TNM rules and 69 (77.5%) sarcomas had a diameter of > 5 cm [21]. The histopathological grade was ≥ 2 in 74 (83.1%) sarcomas. Leiomyosarcoma and Sarcoma NOS were the most common histologic entities (30.3%, respectively 33.7%) (Table 1).

**Table 1:** Characteristics of patients (pts) with soft tissue sarcoma in dependence of sarcopenia

Variables	All pts	Non-sarcopenic pts	Sarcopenic pts	*P*
	*n* = 89 (100%)	*n* = 61 (68%)	*n* = 28 (32%)	
Age at diagnosis STS (years), median (range)	54 (18–79)	52 (18–77)	59 (24–79)	0.055
Age at diagnosis a/mSTS (years), median (range)	60 (20–79)	57 (20–77)	62 (24–79)	0.025
Gender				
Male, *n* (%)	49 (55.1)	35 (57.4)	14 (50)	0.516
Female, *n* (%)	40 (44.9)	26 (42.6)	14 (50)	
Tumor site*				
Superficial, *n* (%)	5 (5.6)	4 (6.6)	1 (3.6)	0.850
Deep, *n* (%)	81 (91.0)	55 (90.2)	26 (92.9)	
NE, *n* (%)	3 (3.4)	2 (3.3)	1 (3.6)	
Size*				
≤ 5 cm, *n* (%)	16 (18)	10 (16.4)	6 (21.4)	0.826
> 5 cm, *n* (%)	69 (77.5)	48 (78.7)	21 (75)	
NE, *n* (%)	4 (4.5)	3 (4.9)	1 (3.6)	
Grading*				
1, *n* (%)	5 (5.6)	5 (8.2)	0 (0)	0.288
≥ 2, *n* (%)	74 (83.1)	49 (80.3)	25 (89.3)	
NE, *n* (%)	10 (11.2)	7 (11.5)	3 (10.7)	
Resection status*				
R0, *n* (%)	45 (50.6)	34 (55.7)	11 (39.3)	0.145
R1, *n* (%)	9 (10.1)	7 (11.5)	2 (7.1)	
R2, *n* (%)	5 (5.6)	3 (4.9)	2 (7.1)	
RX, *n* (%)	10 (11.2)	4 (6.5)	6 (21.4)	
NE, *n* (%)	20 (22.4)	13 (21.3)	7 (25.0)	
Metastatic spread				
Metachronous, *n* (%)	54 (60.7)	36 (59.0)	18 (64.3)	
Synchronous, *n* (%)	35 (39.3)	25 (41.0)	10 (35.7)	0.637
ECOG-Status at diagnosis of a/m STS				
0, *n* (%)	70 (78.7)	47 (77.0)	23 (82.1)	0.586
≥ 1, *n* (%)	19 (21.3)	14 (23.0)	5 (17.9)	
Metastasis at diagnosis of a/m STS				
Lung metastases, *n* (%)	51 (57.3)	37 (60.7)	14 (50.0)	0.345
Liver metastases, *n* (%)	16 (18.0)	10 (16.4)	6 (21.4)	0.566
Lymph node metastases, *n* (%)	16 (18.0)	11 (18.0)	5 (17.9)	0.984
Soft tissue metastases, *n* (%)	17 (19.1)	10 (16.4)	7 (25.0)	0.337
Cerebral metastases, *n* (%)	2 (2.2)	2 (3.3)	0	
Bone metastases, *n* (%)	14 (15.7)	9 (14.8)	5 (17.9)	0.709
Primary site recurrence, *n* (%)	22 (24.7)	13 (21.3)	9 (32.1)	0.271
Other, *n* (%)	15 (16.9)	9 (14.8)	6 (21.4)	0.435
Number of organs with metastasis, median, (range)	1 (1–5)	2 (0–5)	1 (0–5)	0.420
Histologic subtype				0.499
Leiomyosarcoma, *n* (%)	27 (30.3)	17 (27.9)	10 (35.7)	
Liposarcoma, *n* (%)	7 (7.9)	6 (9.8)	1 (3.6)	
Sarcoma NOS, *n* (%)	30 (33.7)	5 (8.2)	2 (7.1)	
Other, *n* (%)	25 (28.1)	19 (31.1)	6 (21.4)	

*At time of primary diagnosis

The median period from initial diagnosis to diagnosis of a/mSTS was 7 (r, 0–207) months, with a predominance of metachronous metastatic spread (*n* = 54, 60.7%), dominated by a pulmonary pattern (*n* = 51, 57.33%) (Table 1).

All 89 patients received a median of 2 (r, 1–8) lines of CTx. Additionally, 52 (58.4%) patients received a multimodal therapy of CTx and either surgery, radiotherapy or both. Overall, patients received a median of 4 (r, 1–11) oncological interventions (Table 2).

**Table 2:** Characteristics of therapy in a/m STS patients (pts) in dependence of sarcopenia

Variables	All pts	Non-sarcopenic pts	Sarcopenic pts	*P*
	*n* = 89 (100%)	*n* = 61 (%)	*n* = 28 (%)	
Number of administered CTx, median (range)	2 (1–8)	2 (1–8)	1.5 (1–7)	0.112
Number of administered Sx, median (range)	0 (0–7)	0 (0–7)	0 (0–4)	0.733
Number of administered RTx, median (range)	0 (0–4)	0 (0–3)	0 (0–4)	0.482
Cumulative no. of therapies, median (range)	4 (1–11)	4 (1–11)	2.5 (1–11)	0.132
MT – (CTx only), *n* (%)	37 (41.6)	22 (36.1)	15 (53.6)	0.122
MT + (CTx + Sx/RTx), *n* (%)	52 (58.4)	39 (63.9)	13 (46.4)	
Medical treatment				
Doxorubicin, *n* (%)	34 (38.2)	25 (41.0)	9 (21.4)	
Doxorubicin + Ifosfamide, *n* (%)	26 (29.2)	20 (32.8)	6 (21.4)	
Other, *n* (%)	29 (32.6)	16 (26.2)	13 (46.4)	
Clinical response (CTx 1)				0.089
CR, *n* (%)	1 (1.1)	0	1 (3.6)	
PR, *n* (%)	20 (22.5)	17 (27.9)	3 (10.7)	
SD, *n* (%)	16 (18.0)	13 (21.3)	3 (10.7)	
PD, *n* (%)	40 (44.9)	24 (39.3)	16 (57.1)	
NE, *n* (%)	12 (13.5)	7 (11.5)	5 (17.9)	
ORR, *n* (%)	21 (23.6)	17 (27.9)	4 (14.3)	0.161
DCR, *n* (%)	37 (41.6)	30 (49.2)	7 (25)	0.032
Treatment discontinued, *n* (%)	58 (65.2)	40 (65.6)	18 (64.4)	0.906
Reason for discontinuation of CTx				0.849
Progression *n* (%)	42 (75.0)	29 (76.3)	13 (72.2)	
Toxicity, *n* (%)	12 (21.4)	8 (21.1)	4 (22.2)	
Other, *n* (%)	2 (3.6)	1 (2.6)	1 (5.6)	

*CTx* chemotherapy, *Sx* surgery, *RTx* Radiotherapy, Cumulative number of therapies: Sum of administered CTx, Sx and RTx, *MT* multimodal therapy, *CR* complete response, *SD* stable disease, *PR* partial response, Mixed: mixed response, *PD* progressive disease, *ORR* objective response rate (CR + PR), *DCR* Disease control rate (CR + PR + SD)

### Patient characteristics in dependence of sarcopenia

The median SMI was 47.7 (26.7–69.6) in men and 40.2 (30.3–64.9) in women. Overall 28 (32%) patients suffered from sarcopenia at diagnosis of a/mSTS (Table 1). No significant difference for gender, tumor stage, tumor grade and tumor localization, etc. was observed in dependence of sarcopenia (Table 1).

At initial diagnosis sarcopenic patients tended to be older than non-sarcopenic patients (p = 0.055) while the age was significantly different at onset of a/mSTS (*P* = 0.025). Sarcopenic patients overall tended to receive fewer oncologic interventions than non-sarcopenic patients (median 2.5, r 1–11 vs. 4, r 1–11, *P* = 0.132), including less lines of CTx (median 1.5, r 1–7 vs. 2, r 1–8, p = 0.112). Also, sarcopenic patients seemed to receive a multimodal treatment approach less often (Table 2).

### Efficacy and outcome of a/mSTS patients in dependence of sarcopenia

Response was numerically higher in non-sarcopenic patients with an ORR of 27.9% compared to 14.3% in sarcopenic patients, but did not reach statistical significance (*P* = 0.161). The DCR of first line CTx was significantly higher in non-sarcopenic patients (49.2% vs. 25%, *P* = 0.032) (Table 2).

**Fig. 2:**
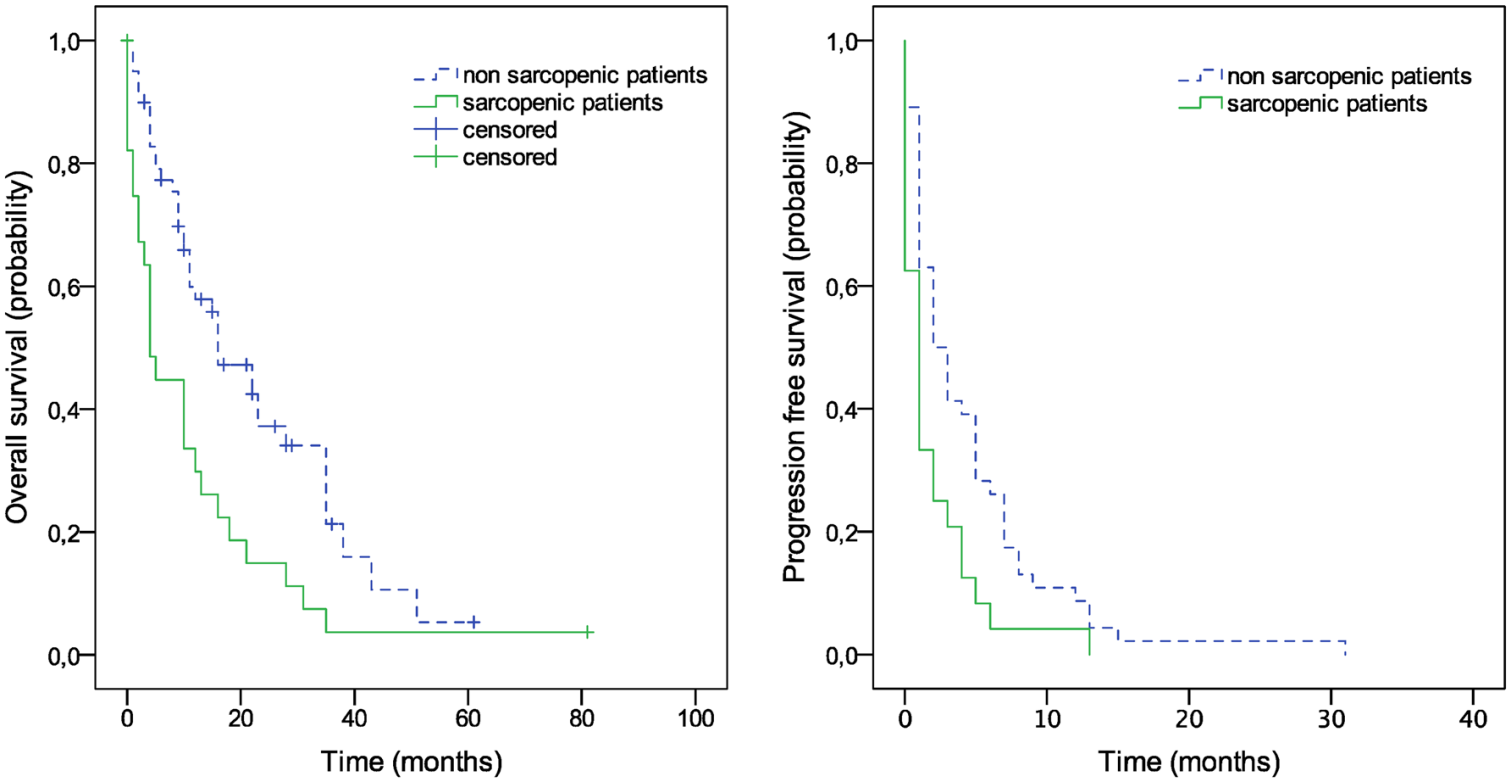
Kaplan–Meier plots. Overall Survival and Progression Free Survival

PFS of first line CTx differed significantly between sarcopenic and non-sarcopenic patients (median 2 months (95% CI, 0.67–3.32), vs. 1 month (95%CI, 0.35–1.65), log-rank *P* = 0.006) (Fig. 2). Median OS of sarcopenic patients was significantly lower compared to non-sarcopenic patients, with 4 months (95% CI, 1.9–6.0) compared to 16 months (95% CI, 8.8–23.2) months in non-sarcopenic patients (log-rank *P* = 0.002) (Fig. 2).

### Sarcopenia as risk predictor for OS and PFS

Univariate analysis for overall survival showed significantly impaired outcome for sarcopenic patients, numbers of involved organ systems by metastasis, as well as a performance status of ECOG ≥ 1 (Table 3). However, subsequent multivariate analysis confirms sarcopenia and ECOG ≥ 1 as independent risk predictors for OS (HR 2.40 [95%-CI, 1.44–4.00], *P* < 0.001; respectively HR 2.34 [95%-CI, 1.36–4.03], *P* = 0.002) (Table 3).

**Table 3:** Cox regression analysis Overall Survival

Variables	Univariable		Multivariable	
	HR (95% CI)	*P*	HR (95% CI)	*P*
Sarcopenia	2.11 (1.28–3,47)	0.003	2.40 (1.44–4.00)	0.001
Age at diagnosis a/mSTS	1.01 (0.99–1.03)	0.392		
Gender	1.63 (0.98–2.73)	0.061	1.66 (.98–2.80)	0.060
Tumor characteristics, at initial diagnosis				
Site (superficial vs. deep)	0.60 (0.27–1.33)	0.209		
Size (≤ 5 cm vs. > 5 cm)	1.02 (0.62–1.68)	0.925		
Grade (G1 vs. G2 + 3)	1.10 (0.64–1.91)	0.725		
Resection status (R0 vs. Other)	1.04 (0.81–1.34)	0.738		
Histologic subtype				
Leiomyosarcoma	1.00	0.470 (df = 3)		
Liposarcoma	1.42 (0.52–3.87)	0.492		
Sarcoma NOS	1.08 (0.57–2.03)	0.815		
Other	1.59 (0.85–2.97)	0.150		
Metastasis at diagnosis a/mSTS (no/yes)				
Lung metastases	0.89 (0.55–1.46)	0.650		
Liver metastases	1.16 (0.64–2.11)	0.631		
Lymph node metastases	1.41 (0.71–2.78)	0.328		
Soft tissue metastases	1.07 (0.57–2.02)	0.833		
Cerebral metastases	5.94 (0.75–47.18)	0.092	4.62 (0.55–38.70)	0.158
Bone metastases	1.41 (0.73–2.71)	0.306		
Primary site recurrence	1.43 (0.80–2.53)	0.227		
Other	1.81 (0.95–3.46)	0.073	1.07 (0.59–1.93)	0.836
No. organs with metastasis (1/ > 1)	1.95 (1.16–3.26)	0.011	1.69 (0.97–2.94)	0.064
Mode of metastatic spread (synchronous vs. metachronous)	0.70 (0.43–1.15)	0.159	0.62 (0.37–1.05)	0.073
MT + (CTx + Sx/RTx), vs. MT – (CTx only)	0.86 (0.52–1.41)	0.538		
ECOG at diagnosis a/mSTS (0/ ≥ 1)	2.33 (1.23–4.38)	0.009	2.34 (1.36–4.03)	0.002

With regard to PFS, sarcopenia and age at diagnosis of a/mSTS were identified as significantly prognostic in univariate analysis. However, multivariate analysis could not confirm these parameters as independent risk factors for PFS of first line medical treatment (Table 4).

**Table 4:** Cox regression analysis progression free survival

Variables	Univariable		Multivariable	
	HR (95% CI)	*P*	HR (95% CI)	*P*
Sarcopenia	1.84 (1.11–3.06)	0.019	1.66 (0.98–2.83)	0.061
Age at diagnosis a/mSTS	1.02 (1.00–1.04)	0.049	1.01 (0.99–1.03)	0.156
Gender	1.28 (0.80–2.07)	0.307		
Tumor characteristics, at initial diagnosis				
Site (superficial/deep)	0.78 (0.30–2.04)	0.611		
Size (≤ 5 cm/ > 5 cm)	0.93 (0.53–1.65)	0.811		
Grade (G1/G2 + 3)	1.17 (0.70–1.95)	0.553		
Resection status (R0/Other)	1.34 (0.76–2.39)	0.315		
Histologic subtype				
Leiomyosarcoma	1 (df = 3)	0.980		
Liposarcoma	0.86 (0.36–2.04)	0.725		
Sarcoma NOS	0.97 (0.53–1.79)	0.922		
Other	0.90 (0.48–1.70)	0.750		
Metastasis at diagnosis a/mSTS (no/yes)				
Lung metastases	0.87 (0.53–1.42)	0.565		
Liver metastases	1.04 (0.62–1.76)	0.879		
Lymph node metastases	1.31 (0.76–2.28)	0.331		
Soft tissue metastases	1.09 (0.65–1.84)	0.742		
Cerebral metastases	1.88 (0.26–13.77)	0.534		
Bone metastases	1.25 (0.69–2.28)	0.467		
Primary site recurrence	1.28 (.76–2.16)	0.357		
Other	1.05 (.62–1.77)	0.863		
No. organs w/metastasis (1/ > 1)	1.37 (0.85–2.22)	0.196	1.35	0.217
Mode of metastatic spread (synchronous/metachronous)	0.89 (0.55–1.45)	0.892		
MT + (CTx + Sx/RTx), vs. MT – (CTx only)	0.76 (0.47–1.24)	0.274		
ECOG at diagnosis a/mSTS (0 / ≥ 1)	1.11 (0.68–1.81)	0.679		

## Discussion

Patients with a/mSTS do have a dismal prognosis, which renders patient counseling a challenge. Up to our knowledge, we report for the first time on the impact of radiologically defined sarcopenia in a cohort of a/mSTS patients prior to palliative medical therapy, which showed a significant association with the outcome parameter OS. Sarcopenia impacted also other efficacy parameter, but independence of sarcopenia as a predictor could not be shown.

89 of 181 a/mSTS patients who received palliative treatment were eligible for this retrospective analysis. This relatively small number is in part owed to the low incidence of soft tissue sarcoma, as well as to the pre-defined inclusion criteria [22]. In particular, the mandated CT-scan within a timeframe of 14 days prior to CTx initiation limited the number of eligible patients. Although our reported cohort of eligible patients is small, it resembles typical characteristics of other STS cohorts, e.g. like previously reported in 78.527 STS patients of the SEER register [23]. Similarities include the important confounder of histology STS subtype distribution, with the most common being sarcoma NOS, leiomyosarcoma and liposarcoma as well as grade ≥ 2 in the majority of patients [23]. Also, age, an important confounder, as well as gender distribution is comparable in both cohorts with a median age of 54 years at primary diagnosis of STS, 58 years at diagnosis of a/mSTS, respectively. In terms of outcome, the median OS of 12 months of the overall cohort is comparable to other recent studies on a/mSTS with similar CTx regimes [24, 25]. The PFS of the overall cohort with a median of 2 months (95%CI 1.40–2.60) in this cohort is lower in comparison to clinical trial results, which is possibly confounded by the real-world population, as illustrated by the performance status, as well as by the selection process for the current study [25]. None the less, all mentioned parameters suggest, to a certain extent, that the analyzed cohort reflects a representative a/mSTS real-world cohort. Although the cohort was quite small, sarcopenia maintained significance as an independent prognostic risk factor, which underlines the strength of this biological stratum.

Stratified for sarcopenia, comparison of the subgroups showed that tumor characteristics between sarcopenic and non-sarcopenic patients mostly do not differ significantly (Table 1). At diagnosis of a/mSTS sarcopenic patients are significantly older (*P* = 0.025). This coincides with other studies. Sarcopenia is highly prevalent in cohorts with solid tumors, reported incidences here vary greatly with ranges from 11 to 74% [26]. It needs to be considered, though, that sarcopenia is also highly prevalent in elderly cancer-free cohorts with percentages ranging from 5 to 50% [27]. Consistent with this, sarcopenic patients show a statistic trend towards being older at initial diagnosis (*P* = 0.055) (Table 1).

With regard to therapy, sarcopenic patients tend to receive less aggressive multimodal therapies than non-sarcopenic patients. Furthermore, there is lower usage of doxorubicin + ifosfamide in sarcopenic patients (41.0 vs. 21.4%), indicating that a less aggressive therapy is more frequently chosen. This supports the assumption that there is already a selection towards less intensive treatment in sarcopenic patients based on clinical judgement. Notwithstanding, the treated sarcopenic patients still tend to profit less from first-line CTx with best clinical response being tumor progression in 57% of cases compared to 39% in non-sarcopenic patients, although the discontinuation rate due to toxicity seemed considerably equal between subgroups. Consequentially, the DCR is significantly lower in sarcopenic patients (*P* = 0.032). Studies on other solid tumors have found an association between sarcopenia and toxicity, and there is another body composition marker, namely the body mass index (BMI), that has been identified as a risk factor for toxicity in a/mSTS [28, 29]. However, this correlation could not be found in our cohort (Data not shown).

Multivariate analysis identified ECOG ≥ 1 and sarcopenia as independent predictors for impaired overall survival. Whether or not, sarcopenia and the performance status describe the same biological phenomenon, or need to be addressed as synergistic parameters was not evaluated by our analysis. However, other studies have found age, gender, histiotype and grade to be prognostic in a/mSTS [9, 10]. With regard to grade, we selected chemo-sensitive STS for medical treatment. This led to inclusion of only 5 patients (5.6%) with grade 1 STS, which renders our study underpowered to perform such a comparison. The finding of ECOG performance status being prognostic is consistent with previous reports in STS [30]. Female gender shows a trend (*P* = 0.060) to have a favorable prognosis, a correlation which is also found in a retrospective review of a large SEER cohort with STS [31].

Most importantly, the CT-derived marker sarcopenia results in a significant difference in OS of 4 months for sarcopenic compared to 16 months of non-sarcopenic patients [HR 2.4 (95-%CI: 1.44–4), *P* = 0.002]. A correlation between sarcopenia and prognosis has already been found in a multitude of different cancer entities, but studies showing this also being applicable to STS are scarce, especially in a setting of a/mSTS. In a study investigating body-composition in STS, sarcopenia did not negatively affect OS [32]. Comparability is limited though, because different to our study, that study investigated localized as well as metastatic disease. In two other analyses with higher proportions of patients with more advanced disease, association of sarcopenia with OS was found [33, 34]. Therefore, the impact of sarcopenia seems to be more significant in a/mSTS compared to localized disease stages and this might be attributed to higher levels of inflammation found in advanced and metastatic disease linked to cancer cachexia [35]. This might contribute to the significant impact of sarcopenia on OS in our cohort of exclusively a/mSTS patients.

Also, we found a significant difference in PFS, albeit small in absolute numbers with 1 month in sarcopenic patients compared 2 months in the non-sarcopenic group. Reflecting the separation potency of sarcopenia, as well as its increasing accessibility, prediction of a/mSTS through SMI seems to be highly interesting in treatment counseling in a/mSTS. However, sarcopenia could not be identified as an independent marker for PFS in our analysis, underscoring its relevance as a prognostic marker.

Although the general definition of sarcopenia as muscle depletion from any cause is widely accepted, as is its principle method of measuring via the CT-derived marker SMI, the variation in reported incidences may partly be explained by the usage of different sarcopenia-defining cut-off values and different methods of measurement in detail [37, 38].

In this study, sarcopenia-defining cut-off values were found by optimal fitting method, the optimal cut-off here being defined as the point with the most significant log-rank test split [20]. The herewith obtained values are close to 2 standard deviations (SD) below the median SMI of a healthy cohort, a method traditionally chosen to find pathology defining cut-off values [39, 40]. There are proposed consensus cut-off values for the SMI available in the literature. However, these consensus cut-off values show a different deviation for men and women compared to a healthy cohort with no apparent explanation [41, 42]. This made the approach of defining cut-off values by optimal fitting method seem more viable and this is also widely practiced [12]. Another possible source of deviation in reported SMI values may be attributed to the use of different software solutions. In this study a self-designed, purpose-built albeit unvalidated software within the MeVisLAB framework is utilized, whereas other studies use commercially available software solutions [33, 34].

Also, this analysis is obviously limited due to its sample size and selection of patients. However, the cohort’s characteristics with regard to demographics and disease resemble those of larger chemo-sensitive STS cohorts.

A limiting factor in using sarcopenia as a marker in routine care is the current technique of measuring. Although CT-scans are routinely performed for staging purposes of STS and thus would be available for body composition analysis, their time-consuming calculation would overly stretch resources of radiology departments in daily care. With the advent of artificial intelligence algorithms, though, it is likely that parameters like the SMI will be among the first that could become readily available as a “byproduct” in routine CT-reports [43].

Further on, comorbidities were not assessed in our analyses, as they were considered negligible in patients with a/mSTS due to their dismal prognosis [36]. However, we cannot rule out a biasing effect of comorbidities in our analyses.

Ultimately, the current finding, that sarcopenia is independently associated with OS, SMI might reflect a powerful tool in prognostication and to some extent in treatment counseling of a/mSTS patients, in terms of choosing aggressive or less aggressive palliative therapy approaches. At length, only prospective trials or lager cohorts will generate higher evidence in relation to this hypothesis.
